# Body-Based Gender Recognition Using Images from Visible and Thermal Cameras

**DOI:** 10.3390/s16020156

**Published:** 2016-01-27

**Authors:** Dat Tien Nguyen, Kang Ryoung Park

**Affiliations:** Division of Electronics and Electrical Engineering, Dongguk University, 30, Pildong-ro 1-gil, Jung-gu, Seoul 100-715, Korea; nguyentiendat@dongguk.edu

**Keywords:** gender recognition, visible light image, thermal image

## Abstract

Gender information has many useful applications in computer vision systems, such as surveillance systems, counting the number of males and females in a shopping mall, accessing control systems in restricted areas, or any human-computer interaction system. In most previous studies, researchers attempted to recognize gender by using visible light images of the human face or body. However, shadow, illumination, and time of day greatly affect the performance of these methods. To overcome this problem, we propose a new gender recognition method based on the combination of visible light and thermal camera images of the human body. Experimental results, through various kinds of feature extraction and fusion methods, show that our approach is efficient for gender recognition through a comparison of recognition rates with conventional systems.

## 1. Introduction

With the development of digital smart systems, such as smart surveillance, or human-computer interaction systems, there are new demands on digital imaging. Many different kinds of information must be obtained in order to perform different system tasks. Information on gender is useful to these smart systems [1,2,3]. For example, the human-computer interaction systems should know gender information in order to respond to users appropriately. The system should respond Mr. or Ms. to users based on gender, and could show specific information to either male or female users. In some other human-computer interaction systems, the systems may need to recognize the individual users and/or their facial expressions in order to interact with them. In this case, gender information can serve as a soft biometrics feature in order to assist recognition results [1,2]. In a surveillance system, knowing the gender information could help the system restrict users from specific restricted areas (the areas are only used by male or female) [3]. The gender information could also be used in demographic collection [1]. Using gender information, the surveillance systems can count the number of males and females who enter a shopping malls or stores. That information is useful for collecting demographic data for commercial purposes [1,2,3].

Because of the many useful applications of gender information, many previous studies have proposed methods for gathering gender information. These previous methods can be grouped into two main categories, the voice-based recognition group [4,5] and image-based recognition group [6,7,8,9,10,11,12,13,14]. In voice-based recognition methods, Mel-frequency cepstral coefficient (MFCC) and pitch values of the human voice are frequently used for gender recognition [4]. High recognition accuracy was obtained by combining the two methods [4]. Although this approach produces high recognition accuracy, noise and microphone quality greatly affects the results.

Gender can also be recognized by using computer vision methods. In this type of method, parts of the human body that contain gender information are captured using camera sensors and then used for gender recognition. In previous researches, images of fingerprints, faces, and/or the entire body were used. In the research of Arun *et al.* [6], fingerprint images are used, and the feature vector describing the ridge thickness to valley thickness ratio (RTVTR) and the ridge density values is obtained. After that, they used the support vector machine (SVM) to classify subjects into male and female groups. Along with fingerprint analysis, computer vision researchers have analyzed gender using facial images. Visually, it is easy to discriminate gender based on the face. Because of this, many previous studies have captured facial images and used them for the gender recognition problem [7,8,9]. In order to enhance recognition accuracy, fingerprint and facial images are combined to solve the gender recognition problem [10,11]. Although the systems using fingerprint, facial, and combined fingerprint-facial images have been proven quite effective for gender recognition problems, they are all limited in that these systems require the cooperation of users when capturing images. Without the cooperation of users, the quality of fingerprint and face images is not sufficient for gender recognition. In addition, in order to capture fingerprint and face images, the system requires a very small distance between the camera sensors and users. This limitation prevents the recognition systems from being applied in surveillance systems.

To overcome the limitations of fingerprint and face-based gender recognition systems, a new biometric modality based on the full body is used. For this reason, the recognition system captures images of the entire body instead of fingerprint and/or face. This approach limits the difficulties presented by low quality of capture images and the cooperation requirement of the fingerprint-based and face-based systems. In the study by Cao *et al.* [12], researchers applied the histogram of oriented gradient (HoG) feature extraction method on body images to extract the image feature. After that, they use the classification method for gender classification. Later, the research by Guo *et al.* [3] enhanced recognition performance by using the biologically-inspired feature extraction method (BIF) to extract image features and used linear support vector machine (SVM) for classification the gender. Through experiments, these studies show that the full body is sufficient for gender recognition up to an accuracy of an about 80% in classification rate using a public database (MIT database). The main limitation of these studies is that they used only visible light images for the recognition problem. The body images have large variation in pose and image texture due to the different clothing and hair styles, accessories *etc.* Consequently, these differences affect the extracted feature and recognition performance. In addition, this method fails if the images are too light or too dark. In that case, the detection of body is firstly failed that causes the wrong gender recognition result. Some other methods use 3-D body-shape for gender recognition [13,14], but in order to obtain the 3-D body-shape, a laser scanner is necessary, and this requires user cooperation, an expensive capturing device, and time for processing. Therefore, this method is hard to apply broadly in surveillance systems since these may require low cost and without cooperation of users. There is no previous research that uses the combination of visible light and thermal images of the full human body for the gender recognition problem. In order to overcome the problem of previous researches on the gender recognition problem, we propose a new gender recognition method that is based on the combination of visible light and thermal images of body. By using the appearance of the full body in both visible light and thermal images, our proposed method captures richer gender information from body for recognition purposes than previous researches in [3,12]. Therefore, the recognition accuracy is enhanced compared to the previous methods that use only visible light images for recognition problem. The research is novel in the following four ways compared to previous studies:

- This is the first study using both the visible light and thermal images of the full human body for gender recognition.
- Based on the detected boxes of body in both visible and thermal images, the features for gender recognition are extracted from the visible light and thermal image. This is accomplished by using the histogram of oriented gradient (HoG) method with principal component analysis (PCA) in order to reduce the feature dimension, processing time, and the effects of noise on the extracted features.
- Gender classification using the features from visible light and thermal images are made by using two different SVMs classifiers.
- A score level fusion is performed to combine the two classification scores by the two SVMs using another SVM classifier in order to recognize the gender of human.

In Table 1, we summarize the previous gender recognition methods and their strengths and weaknesses in comparison to our proposed method. The remainder of this paper is structured as follows: In Section 2, we describe the proposed gender recognition method using the combination of visible light and thermal images of the full human body. In Section 3, we present experimental results using our proposed method applied in a surveillance system. Finally, the conclusions are shown in Section 4.

**Table 1:** Summary of previous studies on image-based gender recognition.

Category	Method	Strength	Weakness
Fingerprint-based gender recognition	Using fingerprint image for gender recognition [6,10,11].	High accuracy	Requires the cooperation of users. The accuracy is affected by quality and the resolution of fingerprint image.
Face-based gender recognition	Using human face for gender recognition [7,8,9,10,11].	High accuracy	Requires the cooperation of users. It’s very hard to recognize gender for very young people.
Body-based gender recognition	Using only visible images of human body for gender recognition [3,12], with 3D shape model [13,14].	Does not require the cooperation of users.	Recognition accuracy is strongly affected by illumination conditions, body poses, the random appearance of image texture on body region such as clothes, accessories *etc.* Recognition accuracy is lower than using face-based gender recognition approach.
	Combining the visible and thermal images of human body using score level fusion using SVM for gender recognition (**Proposed Method**)	Does not require the cooperation of users. Enhances the recognition result compared to the systems that use only visible images for gender recognition. Reduces the affects by illumination condition, the body poses, the random appearance of image texture on boy region such as clothes, accessories *etc.*	Requires longer processing time than singly visible images. Recognition accuracy is still lower than using face-based gender recognition approach.

## 2. Proposed Method

### 2.1. Overview of the Proposed Method

**Figure 1:**
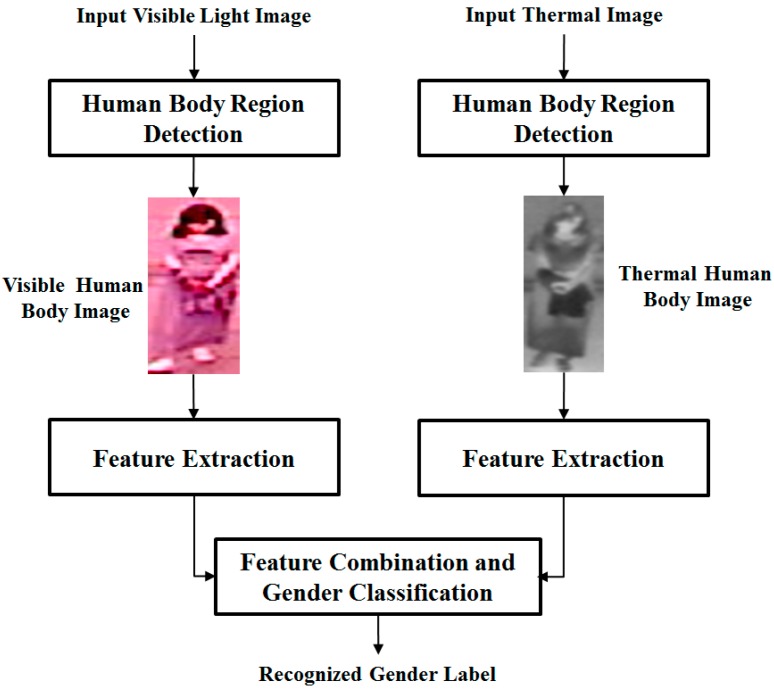
Overall procedure for the proposed method.

In previous studies, it has been shown that images of the human body contain gender information [3,12,13,14]. Based on this information, body images were used for the gender recognition problem. The overall procedure of our proposed method is depicted in Figure 1.

As shown in the figure above, our proposed method uses the appearance of the human body in two different kinds of images, a visible light image and a thermal image for gender recognition purposes. Using visible light and thermal cameras, we capture images that contain corresponding body regions of the same person. The images are first preprocessed using the human detection method [15]. In the second step, our method extracts the image’s features using feature extraction methods such as HoG or multi-level local binary pattern (MLBP). The details of human region detection and image feature extraction will be explained in Section 2.2. After that, the extracted features from the two types of images are combined to construct the final feature for gender recognition. Finally, the gender of users is recognized using SVM.

**Figure 2:**
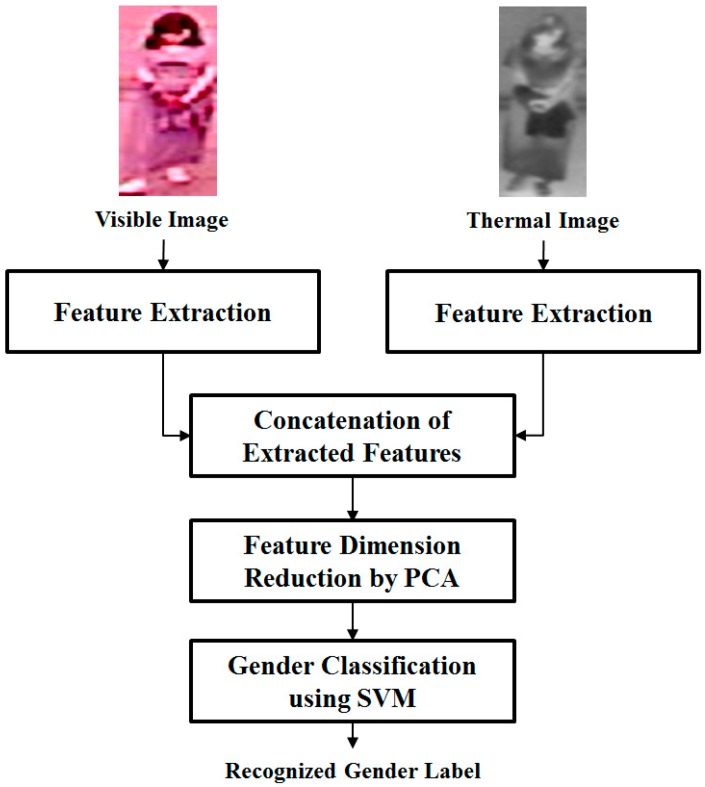
Feature level fusion approach for combining visible light and thermal images.

**Figure 3:**
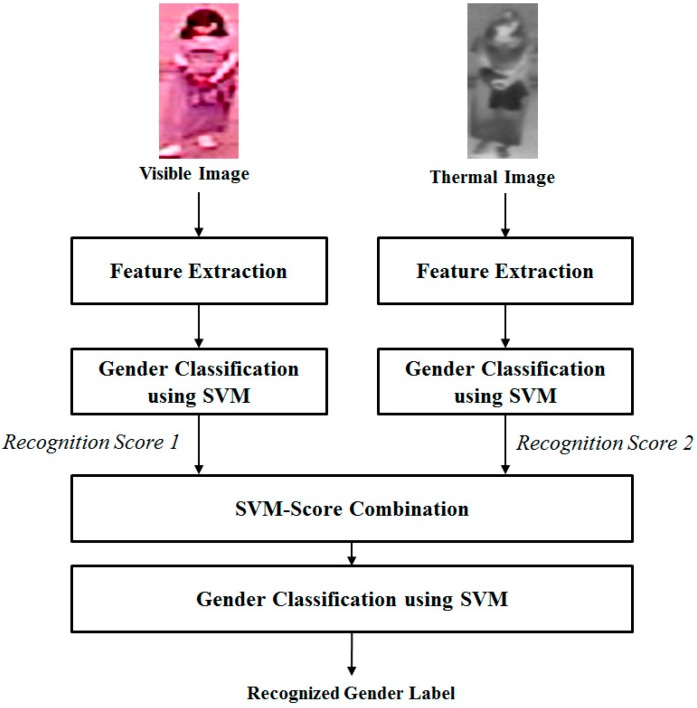
Score level fusion approach for combining the visible light and thermal images.

In order to combine the recognition results of visible and thermal images, our proposed method performs two different combination approaches, feature and score level fusion. The overview of these combination approaches is shown in Figure 2 and Figure 3 for feature level fusion and score level fusion, respectively. As shown in Figure 2, the feature level fusion approach is formed by combining the extracted feature vector of visible and thermal images together. In our research, the combination is performed by concatenating the extracted features of visible and thermal images. Because the feature vectors of the two images are concatenated, the combined feature vector contains the gender information for the two types of images. However, this process causes an increase in feature dimension and associated noise. In order to reduce the feature’s dimension, our proposed method performs a subspace method, based on PCA, on the combined feature [16]. Consequently, we obtain the new feature vector in a lower dimension space than original feature vector. By using PCA, we not only reduce the dimension of the feature vector but also reduce the effects of noise and the processing time of the recognition system. Finally, with the extracted feature (after PCA), we recognize the gender using SVM.

In the second combination approach, we combine the visible and thermal images of the full body using score level fusion as shown in Figure 3. To accomplish this, we first perform gender recognition of visible light and thermal images separately using the first layer of SVM classification.

This layer consists of two SVM classifiers for visible light and thermal images, respectively. For the score level combination, we obtain the decision values of classifiers instead of the predicted class label. The decision values represent the probability that an image belongs to either the male or female class. For example, we assign the male and female classes labels of 1 and −1, respectively. Then, if the decision value is closed to 1, we can think that the image is male. If the decision value is closed to −1, we can think that the image is female. The decision values stand for the probability that an image belongs to one of two classes. Using these first two SVM classifiers, we can obtain two score values (the two decision values). In final step of score level fusion approach, our proposed method uses a second SVM classification layer to recognize the gender using the two input scores from the first SVM classification layer. In our experiments, we will evaluate the recognition performance of a single kind of image (either visible or thermal image) and the two combination methods.

### 2.2. Image Acquisition and Human Detection from Image Sequences

**Figure 4:**
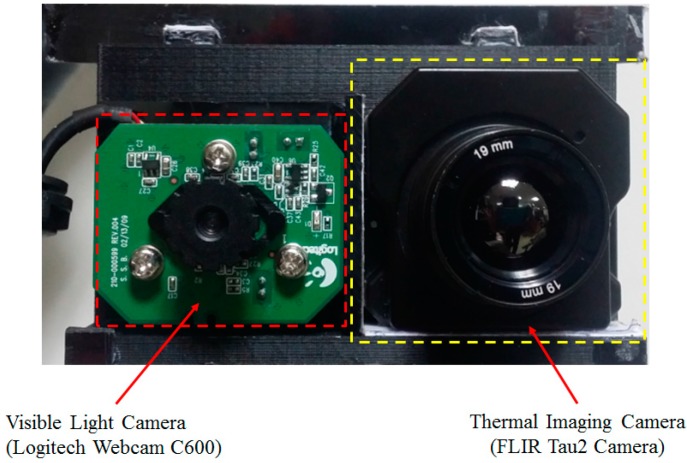
Structure of the dual visible-thermal camera used in our research.

**Figure 5:**
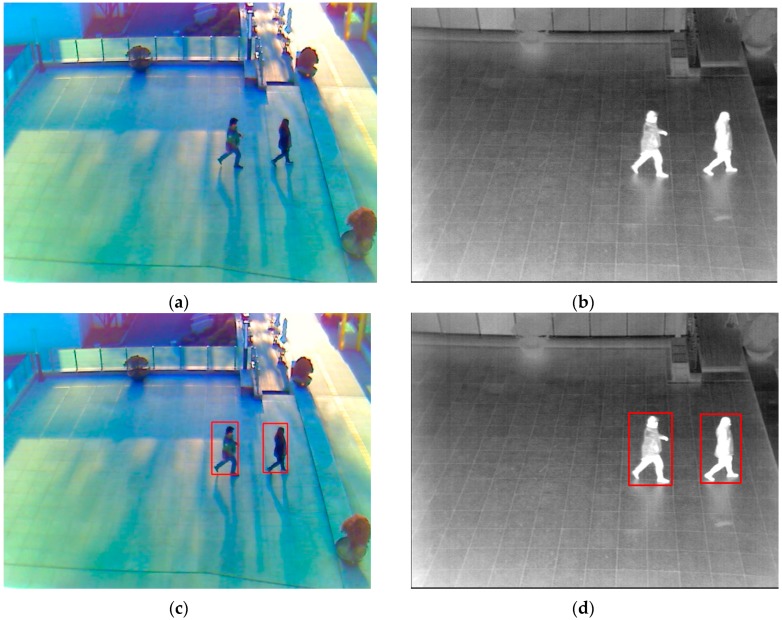
Example of visible and thermal images captured by our lab-made dual visible-thermal camera, and the results of human detection using Lee *et al.*’s method: (**a**) a visible light image; (**b**) a thermal image captured at the same time and same scene with the visible light image; the detection results with (**c**) the visible light image; and (**d**) the thermal image.

In order to recognize gender using body images, we propose capturing visible light and thermal images using a dual visible-thermal camera. A preprocessing step is applied to these images in order to detect the human region and locate the position of the body in these captured images. In our experiment, because there is no public surveillance database that contains both visible light and thermal images, we collect our own database for experiments using our lab-made devices. The structure of our lab-made dual visible-thermal camera is shown in Figure 4. The capturing device in our research consists of two cameras, a visible light camera and a far-infrared light (FIR) camera. We use a low-cost web-camera for the visible light camera since our method is designed for surveillance systems [17]. This camera can capture images with an image resolution of 2 mega-pixels at 30 frames per second (fps). In order to capture the thermal images, either a near-infrared light (NIR) or far-infrared light (FIR) camera can be used. In our research, we use the FIR camera because this kind of camera is more suitable for surveillance systems than NIR camera. The NIR camera can captures signal with wavelengths between 0.75 μm and about 1.4 μm and normally requires an additional NIR illuminators to capture thermal images. This requirement makes the NIR camera ineffective for surveillance systems that need to monitor objects in a far distance, and in an uncontrolled working environment. In contrast to the NIR camera, the FIR camera captures image signal with wavelength range of 8–12 μm and it does not require additional illuminators for capturing images. Therefore, the FIR camera is more appropriate for surveillance applications than the NIR camera. In our lab-made devices, we use the Tau2 [18] commercial thermal camera. In order to combine the two cameras, we attached them on a panel as shown in Figure 4 to fix the position of the cameras in the horizontal direction and reduce the disparity between the two cameras. In Figure 5a,b we show some examples of images captured by our lab-made dual visible-thermal camera.

With the captured visible and thermal images, our proposed method performs a preprocessing step to extract the body images in both images. To do this, we use the method proposed by Lee *et al.* [15] for human detection. Using this method on both images, we enhance the detection accuracy when compared to using a single type of image. In Figure 5c,d, we show some examples of detection results using Lee *et al.*’s method [15]. As shown in these figures, the region of interest (ROI) of the human body is well located in the captured visible and thermal images. Using this result, we extract the body images and use them for gender recognition in the later steps of our proposed method.

### 2.3. Feature Extraction Methods for Gender Recognition

After identifying body boxes within images, the next step in our proposed method is to extract image features from the images. The simplest way to use these images is to use the raw pixels directly. However, direct use of the raw pixels is not a good choice due to the large variation in these images such as clothing color, illumination change, and hairstyle. In order to extract the more distinct gender features for gender recognition purpose, we use two different feature extraction methods. We use the HoG and MLBP methods to evaluate the performance of recognition system for inter-experimental comparison.

#### 2.3.1. Histogram of Oriented Gradient

The HoG feature extraction technique was successfully applied on the human detection problem [19] and the gender recognition problem [12]. In previous research on gender recognition by Cao *et al.* [12], the HoG technique was firstly used on the problem and had good performance. As indicated by its name, the HoG feature extraction method extracts image features by collecting the magnitude-weight and direction of edge features in sub-blocks of an image. Consequently, we can obtain a map of age of the entire image. For our problem, the gender information is mainly measured based on body-shape and the different kinds of edge in a body image. Therefore, the HoG feature technique is efficient for gender recognition as indicated in [12]. In our studies, we use the HoG technique for extracting image features for the gender recognition problem.

#### 2.3.2. Multi-Level Local Binary Pattern

As mentioned in previous sections, the human body images-based gender recognition problem is complicated by illumination variations in the captured body images. Previous studies in computer vision have proven that the local binary pattern (LBP) is a powerful image feature extraction method. The main advantage of the LBP is that it is invariant to the illumination levels of an image. Based on this characteristic, the LBP has been successfully used for many biometrics systems such as finger-vein recognition [20], face recognition [21], age estimation [16,22,23], gender recognition [24], and face re-identification [25]. In previous research on human age estimation, the author proposed the use of a multi-level LBP methodology instead of simple LBP for age estimation problem [16,22]. By accumulating the histogram of uniform and non-uniform LBP features in an image, we can identify features such as lines (edges), corners, spots *etc.* This methodology yielded good results for age prediction. In addition, the MLBP feature (image feature extracted by MLBP method) has been proven to outperform the LBP feature [16,22]. Inspired from this research, we use MLBP feature extraction method for the gender recognition problem. The LBP operator is mathematically defined in Equation (1):

(1) $$LBP_{P,R}=\;\sum_{p=0}^{P-1}s\left(x_{c}-x_{p}\right)\times 2^{p},\mathrm{where}s\left(x\right)=\{\begin{array}{c} 0\;if\;x<0\\ 1\;otherwise \end{array}$$

In Equation (1), R value indicates the radius of a circle that the surrounding pixels are located within, and P is the number of surrounding pixels. As shown in this equation, we see that an illumination invariant characteristic is obtained using the LBP method. By comparing the center pixels with its P surrounding pixels, the LBP feature extraction method works as an adaptive thresholding method for extracting the image texture feature. Consequently, a LBP code of P bits is obtained to encode each pixel in an image. Although the illumination conditions could be changed by changing image-capturing conditions, the LBP method offers the same image features regardless of illumination. By using various values of R and P, we can extract the image feature at different scales and resolutions. In order to construct the image feature, the extracted LBP codes are first divided into uniform and non-uniform codes [16,22]. The uniform codes are defined as containing at most two bit-wise transitions from 0 to 1 (or 1 to 0). The other kinds of codes are defined as non-uniform code. For example, 00000000 and 011111000 are uniform codes because they contain two bit-wise transitions from 0 to 1 (or 1 to 0), while 01010100 and 01100110 are non-uniform codes because they contains six and four bit-wise transitions, respectively. The uniform codes contain useful texture information such as edge, corner, spot *etc.* On the other hand, the non-uniform code describes very complex image texture features. These complex textures could also be caused by noise. Therefore, this kind of texture feature is not sufficient for making an image feature. Similar to the age estimation problem, we see that the direction of the texture (edge, corner *etc.*) is not as important for the gender recognition problem as the appearance and the number of texture feature in image. Therefore, we further group the uniform LBP codes by considering patterns that have similar texture shape but could be going in different directions. By performing this step, we make the rotation invariant LBP code [16,22]. Based on that, we assign each group of uniform rotation invariant texture patterns a specific decimal code from 0 to P, while all the non-uniform patterns are grouped together and assign a decimal code of (P + 1). With the assigned decimal codes, we can easily create a histogram of the appearance of texture features and use them for the gender recognition problem.

**Figure 6:**
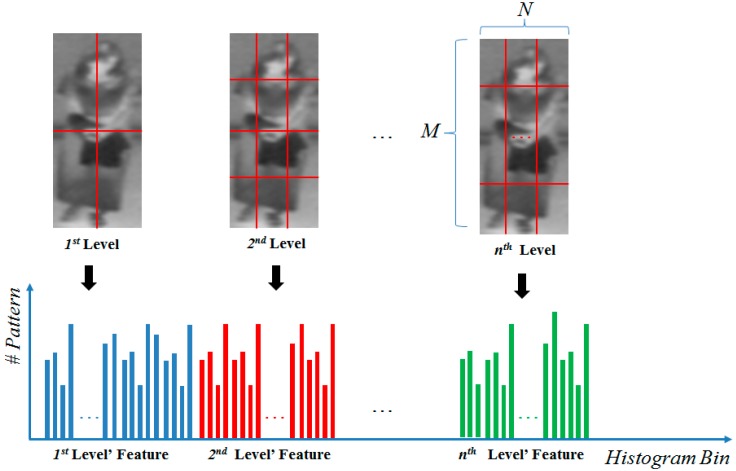
Method for extracting the MLBP feature from an image.

In order to extract image features more efficiently, previous researchers [16] divided the image into sub-blocks and constructed the image feature by concatenating the extracted features of each sub-block. This scheme was successfully applied for face-based human age estimation [16,22] and the extracted feature is called a single level LBP feature. By using this method, the extracted image features not only contain the global image feature but also the local image feature. As reported in previous research, the MLBP feature outperforms the single level LBP feature. In Figure 6, we demonstrate methodology of MLBP feature extraction method. In this figure, *M* and *N* are the number of sub-blocks in the vertical and horizontal directions. By combining several single level LBP features, we form the MLBP feature for the gender recognition problem.

#### 2.3.3. Feature Combination and Gender Recognition Using SVM

The final steps of our proposed method are the combination of features from the visible light and thermal images and the recognition of gender. As shown in Section 2.1 and Figure 2 and Figure 3, the combination of visible and thermal images can be performed using either the feature level fusion or score level fusion approach. In feature level fusion, the two extracted features are concatenated together to form the final feature for gender recognition as shown in Equation (2)

*f* = [*f_1_*, *f_2_*] (2)

By concatenating the two features, the combined feature can contain the gender information of both the visible light and thermal images. In Equation (2), the combined feature *f* is obtained by combing two features (*f_1_* and *f_2_*) which are extracted using the visible and thermal image. In the second combination approach, gender classification using visible light and thermal images is performed separately using two SVM classifiers. Using the two SVM classifiers, we obtain the scores for gender classification (decision values of SVM classifiers) with visible and thermal images. These scores are concatenated and input into the third SVM classifier for the score level fusion approach as shown in Figure 3.

The features inputted to the SVM classifiers must be normalized. To do this, we use Z-score normalization method as shown in Equation (3) [16]. In this equation, μ and σ indicate the mean and standard deviation of features in the training database. By using the mean and standard deviation as shown in Equation (3), we normalize the feature and make it similar to the normal distribution:

(3) $$f=\frac{f-\mu }{\sigma }$$

Features extracted by HoG, or MLBP, or feature level fusion have high dimensional characteristic. This characteristic causes difficulty in the classification step based on SVM. In order to solve this problem, our proposed method performs an additional preprocessing step using principal component analysis (PCA). The PCA is a well-known method for dimension reduction and has been used in previous computer vision applications [16]. To do this, we first calculate the covariance matrix *C* using the extracted image features from the training images using Equation (4):

(4) $$C=\frac{1}{N}\overset{N}{\underset{n=1}{\sum }}\left(x_{n}-\mu \right){\left(x_{n}-\mu \right)}^{T}$$

where, *N* indicates the total number of training samples, *x_n_* is the *n^th^* extracted image feature, μ is the mean value of extracted features, and *T* is the transpose operator. Using the covariance matrix *C*, we obtain a transformation matrix *W* by using *n* eigen-vectors that correspond to the *n* largest eigen-values of *C*. The number of eigen-vectors is normally smaller than the dimension of original feature and determined through experiments in which the gender recognition performance is highest. Then, this transformation matrix (*W*) is used to transform a new input image feature into a low-dimensional feature space [16].

In the final step of our proposed method, we recognize the gender using the SVM method. To do this, we use the OpenCV (ver. 2.4.9) library [26]. SVM classifies input images into male and female classes using support vectors and kernel function. In the general case, the SVM is described as Equation (5). In Equaiton (5), *f(x)* indicates the classification function; *x_i_* is the support vector; and *K()* is the kernel function that is used to transform the input data into a higher dimensional space. In our experiment, we use two kinds of SVM kernels, linear and RBF, as shown in Equations (6) and (7):

(5) $$f\left(x\right)=sign(\overset{k}{\underset{i=1}{\sum }}a_{i}y_{i}K\left(x,x_{i}\right)+b)$$

(6) $$\mathrm{Linear kernel}:\;K\left(x_{i},x_{j}\right)={x_{i}}^{T}x_{j}$$

(7) $$\mathrm{RBF kernel}:\;K\left(x_{i},x_{j}\right)=e^{-\gamma {\left\|x_{i}-x_{j}\right\|}^{2}}$$

The linear kernel is the simplest kernel and normally used to perform the linear classification problem. And, the RBF kernel is a general kernel that is suitable for most classification problems. Using the training dataset, we can obtain the support vectors as well as the kernel parameters. These support vectors and kernel’s parameters are saved and used for gender classification.

## 3. Experimental Results

### 3.1. Description of the Database, Performance Measurement and Experimental Setup

**Figure 7:**
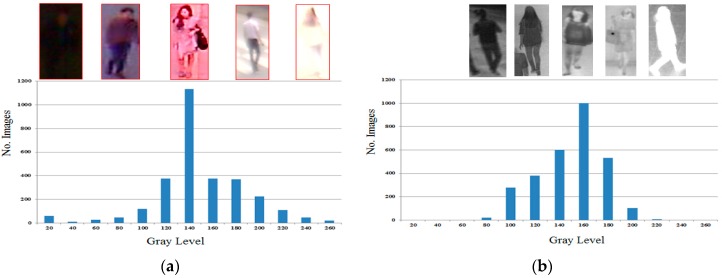
The distribution of illumination of images in our collected database: (**a**) illumination distribution of visible light images; and (**b**) illumination distribution of thermal images.

Because there is no public database that contains both visible light and thermal images of the human body, for our experiments we collected our own database using our lab-made device (Figure 4). The database consists of 5852 images of both visible light and thermal images of the human body from 103 persons (66 males and 37 females) with differences in background and body-pose (front, back, side pose). In order to simulate the application of our proposed method in surveillance systems, the dual-camera (in Figure 4) was placed at a height of about 6 m. We captured several images of each person at different times to simulate the effects of body pose. On average, we captured 28 images for each person in different poses. All the images (visible light and thermal images) were captured in an un-controlled environment. Therefore, the illumination and environment greatly affects the captured images. In details, we capture images in both the daytime and nighttime to vary illumination strength. In Figure 7, we show the distribution of image illumination in the visible light and thermal images of our collected database. In previous researches, only the visible images are used for gender recognition [3,12]. Therefore, if the captured image is too dark or too light, detection of the body region could fail and the recognition performance decreases. In this case, the use of thermal images complements the use of visible images. Because the thermal camera captures images using infrared light that is radiated due to the body temperature, the appearance of full body in thermal images is normally distinguished from the background even if the illumination condition is poor (too dark or to bright). As shown in Figure 7, the illumination of visible images in our database contains a large spread from too dark too bright; whereas the illumination of the thermal images is in a smaller brightness range.

**Figure 8:**
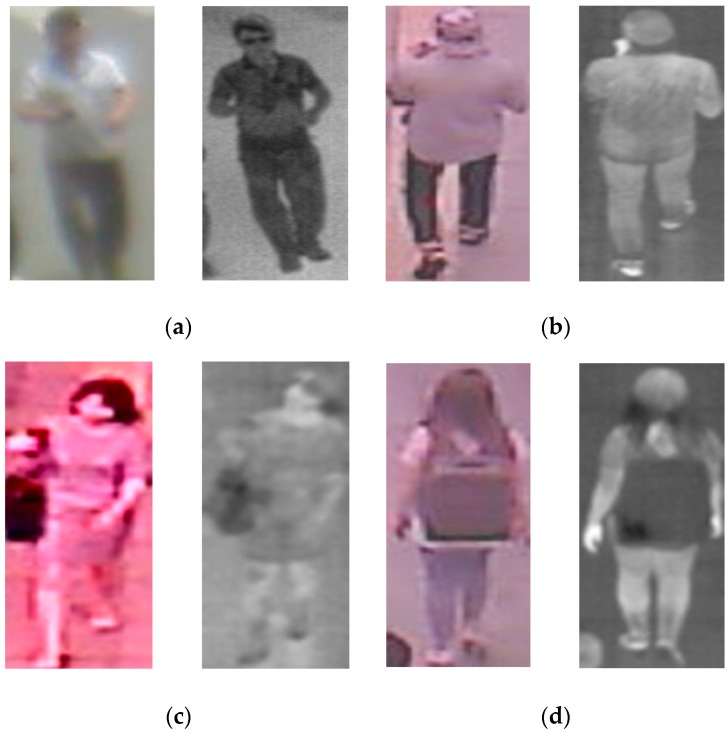
Examples of visible light and thermal image pairs in our collected database: (**a**) visible (left)—thermal (right) images of a male in front view; (**b**) visible (left)—thermal (right) images of a male in back view; (**c**) visible (left)—thermal (right) images of a female in front views and (**d**) visible (left)—thermal (right) images of a female in back view.

We randomly divided the database into learning and testing sub-databases five times in order to perform a 5-fold cross-validation scheme. In details, the images (both visible light and thermal images) of 53 males and 30 females are used for training, and the remaining images of 13 males and 7 females are used for testing. A detailed description of our collected database is given in Table 2 and Table 3. In order to evaluate the recognition accuracy of our proposed method, we measure the equal error rate (EER) of the training and testing database. By measuring the recognition accuracy of each training-testing time, we obtain the five EER values and the final recognition result of the database is measured as the average value of the five obtained EERs values. The EER is a popular criteria used in biometric systems that indicates the boundary of the recognition system where the false acceptance rate (FAR) is equal to false rejection rate (FRR). For the gender recognition problem, we have two classes of male and female. The FAR value indicates that a female image is falsely recognized as male images; and the FRR value indicates that a male image is falsely recognized as a female image. In Figure 8, we show some examples of visible light and thermal images of the full body that are extracted from our database using the human detection method proposed by Lee *et al.* [15].

**Table 2:** Description of visible light and thermal images database of the human body in our experiments.

Database		Visible Database	Thermal Database	(Visible + Thermal) Database
Number of persons	103 (66 males/37 females)		103 (66 males/37 females)	103 (66 males/37 females)
Number of Images	2926		2926	5852

**Table 3:** Description of learning and testing sub-databases.

Database	Training Sub-Database	Testing Sub-Database	The Entire Database
Number of persons	83 (53 males/30 females)	20 (13 males/7 females)	103 (66 males/37 females)

### 3.2. Gender Recognition Using Our Proposed Method

In our first experiment, we measure the recognition accuracy of the recognition system using a single kind of image, either visible light or thermal. When using a single kind of image, the combination step is removed from Figure 1. In this case, the recognition method performs human detection, feature extraction, and recognition using SVM. The use of only visible images corresponds to a conventional system using only visible light images for recognition purpose as in previous studies [3,12]. In addition, by using the thermal images of full human body, our experiments also evaluate the suitability of thermal images for the gender recognition problem. For comparison purposes, we perform experiments using two kinds of feature extraction method as mentioned in Section 2, HoG and MLBP, two kinds of SVM kernels, linear and RBF. The experimental results are shown in Table 4. Also shown in Table 4, the best system performance using only visible images was obtained using the RBF kernel, with EERs of 16.540% (HoG) and 25.088% (MLBP), respectively. Similarly, we obtained the best accuracies (EER) of 19.583% and 20.572% by using the HoG and MLBP feature extraction methods, respectively, using only thermal images. In addition, the recognition accuracy of systems that use the linear kernel of SVM is lower than those that use the RBF kernel. Through these experimental results, we can conclude that the RBF kernel is better suited to gender recognition than the linear kernel. However, the use of the RBF kernel is more complicated than the use of linear kernel, and takes a longer processing time for training and testing than the linear kernel.

As shown in Table 4, the HoG feature extraction method works better than the MLBP feature extraction method by producing lower EER values for both visible and thermal images. This is due to the characteristics of body images and the features extracted by each method. Because the body images have a large variation in poses, hairstyle, clothing and accessories, the gender information is mostly extracted by shape of the body and/or body’s parts. The HoG feature extraction method extracts the strength and the direction of edges on image’s blocks. Therefore, it is efficient for describing the body shape. MLBP on the other hand, extracts the statistical characteristics of the appearance of texture features in an image such as an edge, corner, or spot *etc.* Due to the variation in body images, the appearance of detailed texture features varies for each individual. Consequently, noise has a greater effect on the MLBP technique than HoG feature extraction.

**Table 4:** Accuracies of recognition system (EER, FAR *vs.* GAR) that uses only visible or thermal images of body for gender recognition (the values of GAR and FAR at the EER position are shown in bold-type) (unit: %).

Methods		Linear Kernel			RBF Kernel		
		EER	FAR	GAR	EER	FAR	GAR
Using only visible images for recognition	Using HoG feature	19.639	10.000	58.099	**16.540**	10.000	62.758
			15.000	69.920		15.000	79.878
			**19.642**	**80.364**		**16.542**	**83.461**
			20.000	80.440		20.000	88.051
			25.000	85.697		25.000	91.716
	Using MLBP feature	27.105	20.000	62.790	25.088	15.000	57.506
			25.000	69.676		20.000	66.359
			**27.107**	**72.897**		**25.094**	**74.918**
			30.000	77.793		30.000	80.778
			35.000	81.359		35.000	86.088
Using only thermal images for recognition	Using HoG feature	23.459	15.000	60.002	**19.583**	10.000	57.027
			20.000	67.192		15.000	65.791
			**23.518**	**76.600**		**19.605**	**80.439**
			25.000	79.049		20.000	80.669
			30.000	83.499		25.000	89.815
	Using MLBP feature	24.002	15.000	61.901	20.572	10.000	55.929
			20.000	68.824		15.000	68.506
			**24.019**	**76.014**		**20.580**	**79.436**
			25.000	76.771		25.000	85.236
			30.000	81.354		30.000	90.097

Based on the extracted features using visible light and thermal images of human body, in our next experiments we perform the combination of visible and thermal images for gender recognition as shown in Figure 2 and Figure 3. In our second experiment, we combine the two kinds of images using the feature level fusion approach as shown in Figure 2. To do this, the extracted features in each image are concatenated together to form the final feature that contains the gender information in both images. The experimental results are given in Table 5 using the HoG and MLBP feature. Using the HoG feature, the combination of the two kinds of images produce an EER of 15.946%, which is smaller than the EER of 16.540% produced when using only visible images, or EER of 19.583% when using only thermal images. Using the MLBP feature, the recognition accuracy is reduced from 25.088% using visible images, or 20.572% using thermal images, to 18.126% through the feature level fusion approach. These results demonstrate that the combination of two kinds of images can help enhance the accuracy of the recognition system.

**Table 5:** Recognition accuracy (EER, FAR *vs.* GAR) of the recognition system using feature level fusion for combining visible and thermal images for gender recognition (the values of GAR and FAR at the EER position are shown in bold-type) (unit: %).

Method	Linear Kernel			RBF Kernel		
	EER	FAR	GAR	EER	FAR	GAR
Using HoG feature	19.553	10.000	61.445	**15.946**	10.000	73.050
		15.000	70.169		15.000	80.534
		**19.605**	**80.498**		**16.192**	**84.299**
		20.000	80.790		20.000	89.341
		25.000	84.764		25.000	92.250
Using MLBP feature	21.022	15.000	63.366	18.126	10.000	60.993
		20.000	76.482		15.000	73.669
		**21.043**	**78.999**		**18.155**	**81.902**
		25.000	83.096		20.000	84.426
		30.000	87.075		25.000	89.898

In the third experiment, we evaluate the recognition performance of the recognition system using the score level fusion approach as depicted in Figure 3. As indicated in Section 2.1, the score level fusion approach requires two layers of SVM classification. The first layer is used to classify the subject gender using the visible light and thermal images separately. This layer outputs scores that indicate the probability of an image belonging to male or female classes. In the second layer, the gender is finally recognized using the scores obtained from the first layer. The detailed results for the HoG and MLBP feature extraction methods are given in Table 6.

As shown in the table, the best accuracy is obtained using the HoG feature extraction method and the RBF kernel in all layers of SVM classification. The EER for the score level combination approach is 14.672%, which is smaller than the errors when using just visible light or thermal images alone (16.540% and 19.583% in Table 4, respectively) or even the feature level fusion approach (15.946% in Table 5). Using the MLBP feature extraction method, the score level fusion produces an EER of 17.642%, which is smaller than the errors produced using single solely visible light or thermal images (25.088% and 20.572% in Table 4, respectively) and the feature level fusion approach (18.126%). Through the experimental results in Table 4, Table 5 and Table 6, we can conclude that the combination of visible and thermal images can help a recognition system enhance its accuracy. In addition, the score level fusion approach outperforms the feature level fusion approach by producing lower recognition error than feature level approach. In Table 7, we summarize the recognition results of our experiments. As shown in this table, the score level fusion method using the HoG feature and RBF kernel (in both layers of SVM classification) produced the best recognition result.

**Table 6:** Recognition accuracy (EER, FAR *vs.* GAR) of the recognition system using score level fusion for combining visible and thermal images for gender recognition (the values of GAR and FAR at the EER position are shown in bold-type) (unit: %).

Feature Extraction Method	The 1st SVM Kernel	The 2nd SVM Kernel	Recognition Accuracy		
			EER	FAR	GAR
Using HoG feature	Linear	Linear	17.891	15.000	75.063
				**17.892**	**82.111**
				20.000	84.353
				25.000	88.862
		RBF	17.628	15.000	75.143
				**17.667**	**82.411**
				20.000	84.696
				25.000	89.035
	RBF	Linear	15.158	10.000	65.884
				15.000	83.988
				**15.254**	**84.937**
				20.000	93.900
		RBF	**14.672**	10.000	71.395
				**14.754**	**85.410**
				15.000	85.410
				20.000	94.422
Using MLP feature	Linear	Linear	20.835	15.000	71.802
				20.000	76.199
				**21.068**	**79.397**
				25.000	83.019
		RBF	20.755	15.000	63.043
				20.000	76.298
				**21.068**	**79.559**
				25.000	84.419
	RBF	Linear	17.857	10.000	59.592
				15.000	75.281
				**17.892**	**82.178**
				20.000	85.796
		RBF	17.642	10.000	57.442
				15.000	75.895
				**17.667**	**82.383**
				20.000	86.253

This result proves that our proposed method is efficient for gender recognition in surveillance systems. In addition, our proposed method outperforms the conventional method that uses single kind of body images (only visible or thermal images) for recognition problem [12].

**Table 7:** Summary of recognition accuracy (EER, FAR *vs.* GAR) using a single image type verses the combination of visible and thermal images (the values of GAR and FAR at the EER position are shown in bold-type) (unit: %).

Visible Light Image (Conventional Method Using Single Visible Light Images)			Thermal Image (Conventional Method Using Single Thermal Images)			(Visible Light + Thermal) Images					
						Feature Level Fusion			Score Level Fusion (Proposed Method)		
EER	FAR	GAR	EER	FAR	GAR	EER	FAR	GAR	EER	FAR	GAR
16.540	10.000	62.758	19.583	10.000	57.027	15.946	10.000	73.050	**14.672**	5.000	62.693
	15.000	79.878		15.000	65.791		15.000	80.534		10.000	71.395
	**16.542**	**83.461**		**19.605**	**80.439**		**16.192**	**84.299**		**14.754**	**85.410**
	20.000	88.051		20.000	80.669		20.000	89.341		15.000	85.410
	25.000	91.716		25.000	89.815		25.000	92.250		20.000	94.422

**Figure 9:**
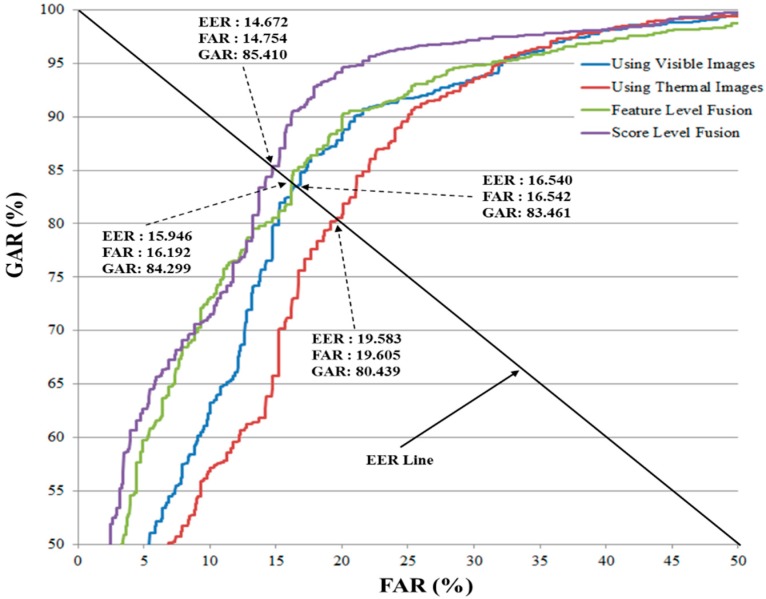
The average ROC curve of recognition systems using different kinds of images.

In Figure 9, we show the receiver operating characteristic (ROC) curve for the recognition systems using a single kind of visible/thermal image, feature level fusion, and score level fusion approaches. In this figure, the GAR is the genuine acceptance rate and it is defined as (100–FRR) (%). The figure demonstrates the superior performance of score level fusion methodology when compared to the other investigated methods.

**Figure 10:**
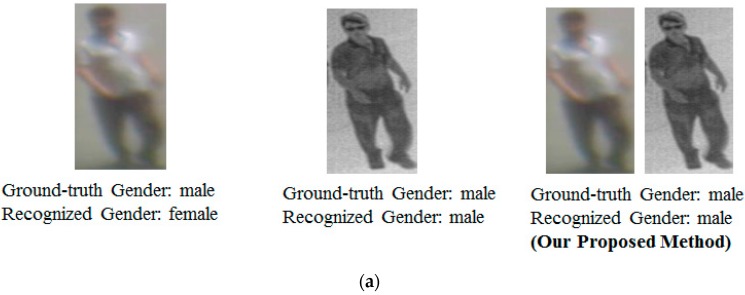

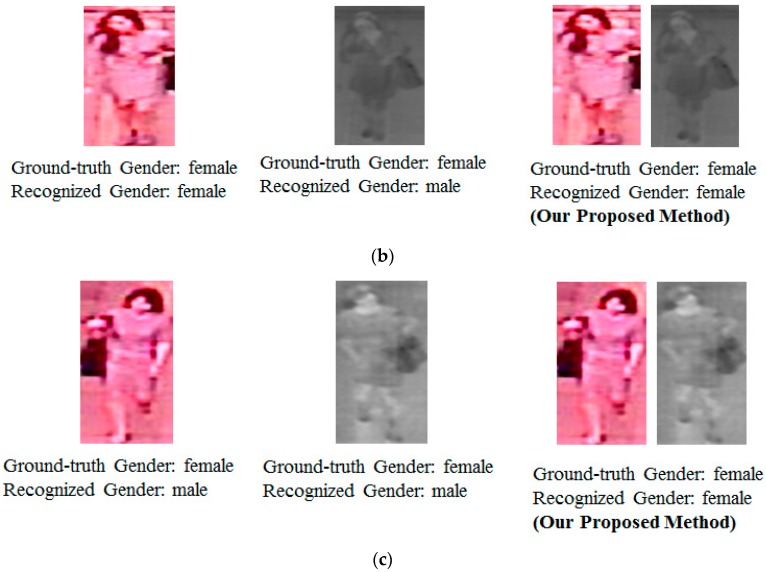
Examples of recognition result using our proposed method: (**a**) recognition result of a male; (**b**,**c**) recognition results of females.

**Figure 11:**
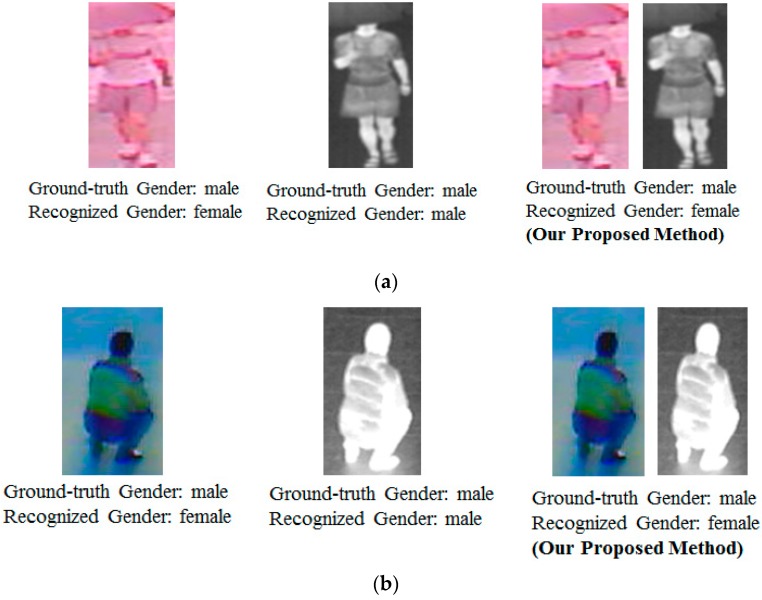

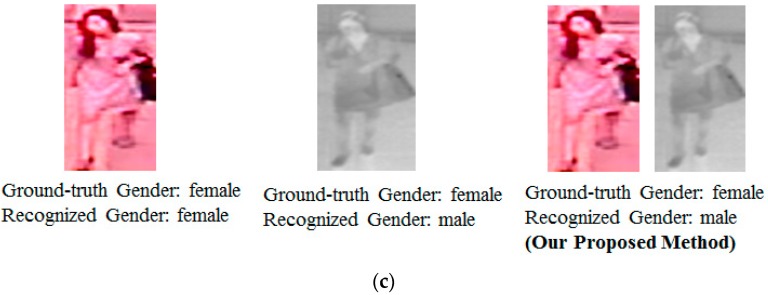
Examples of poor recognition results using our proposed method: (**a**) images with occlusion; (**b**) images with un-normal body-pose; and (**c**) images with poor quality.

For demonstration purpose, we also show some examples of recognition results using our proposed method against a system that uses only visible and thermal images for the recognition problem in Figure 10 and Figure 11. As shown in Figure 10, although recognition using single images of visible and/or thermal images are fail, the combination of the two kinds of images produce better recognition results. However, the combination still recognizes incorrectly when the input images are too poor in quality, are accompanied by a shadow, or un-normal human body pose as shown in Figure 11.

In the next experiment, we measure the processing time of our proposed method. The results are summarized in Table 8. This experiment was performed using a desktop computer with an Intel Core i7 CPU (3.5 GHz) with 8 GB of RAM. Averagely, it took about 27.5948 ms to process two visible and thermal images and to produce the recognition result. Therefore, we conclude that our proposed method can work in real-time at a speed of up to 36 frames per second (1000/27.5948).

**Figure 12:**
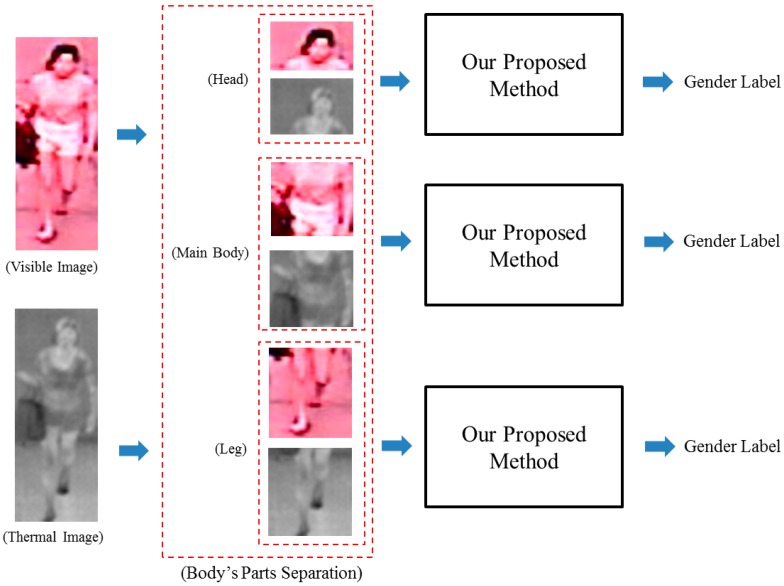
Procedure of the experiments to evaluate the gender recognition ability of different body parts.

Finally, we use our proposed method to evaluate gender based on only parts of the body. To do this, we divided the entire human body image into three parts: The head, main body, and leg. After that, the recognition accuracies were evaluated using each part of the images. Figure 12 shows the procedure of these experiments.

**Table 8:** Processing time of our proposed method (unit: ms).

Human Body Detection	Feature Extraction Using HoG (Two Images)	Feature Dimension Reduction (PCA) (Two Images)	The 1st SVM Layer	The 2nd SVM Layer	Total
23.1300	1.6335	2.7548	0.0700	0.0065	27.5948

The main purpose of these experiments is to exploit the gender information contained in different parts of the human body. Based on previous experimental results, we see that our proposed method produced the best recognition accuracy using the score level fusion method with the RBF kernel. Therefore, in these experiments, we use this fusion approach and the RBF kernel to measure the recognition accuracy of the head, main body and legs. The detail recognition accuracies are shown in Table 9. As shown in Table 9, the head part produces the best recognition accuracy (EER of 15.620%) compared to those of main body part (EER of 20.386%) and leg part (EER of 22.591%) when using the HoG feature extraction method. In addition, the combination of visible and thermal images outperforms the use of solely single visible or thermal images for the purpose of gender recognition. From these results, we can conclude that the parts of head and main body contain more gender information compared to the leg part of human body. The reason is that the leg part contains considerable background noise and limited gender information, such as hair or clothing style when compared to the head or main body parts. Consequently, the recognition accuracy is low and substantially affected by noise compared to other parts of the body.

**Table 9:** The gender recognition performance (EER, FAR *vs.* GAR) of different parts of human body using our proposed method (the values of GAR and FAR at the EER position are shown in bold-type) (unit: %).

Body’s Part	Head Part			Main Body Part			Leg Part		
	EER	FAR	GAR	EER	FAR	GAR	EER	FAR	GAR
Using Visible Image	18.238	10.000	65.670	25.788	20.000	64.472	25.175	20.000	66.787
		15.000	75.548		25.000	73.242		25.000	74.298
		**18.242**	**81.767**		**25.794**	**74.217**		**25.256**	**74.906**
		20.000	83.931		30.000	79.404		30.000	79.806
		25.000	88.913		35.000	85.630		35.000	84.513
Using Thermal Image	22.779	15.000	66.376	26.845	20.000	62.066	27.414	20.000	61.674
		20.000	71.566		25.000	69.666		25.000	69.402
		**22.856**	**77.298**		**26.982**	**73.291**		**27.419**	**72.592**
		25.000	82.200		30.000	77.203		30.000	75.261
		30.000	87.841		35.000	83.998		35.000	78.912
**Our Proposed Method**	**15.620**	10.000	70.668	**20.386**	15.000	65.923	**22.591**	15.000	67.983
		15.000	83.506		20.000	76.988		20.000	73.893
		**15.679**	**84.440**		**20.580**	**79.808**		**22.631**	**77.448**
		20.000	88.997		25.000	84.182		25.000	79.688
		25.000	92.181		30.000	88.564		30.000	84.863

## 4. Conclusions

In this paper, we have proposed a gender recognition method using a combination of visible and thermal images of the human body. Due to the variations in image capturing conditions and the random appearance of the subject due to clothes, accessories, *etc.* the recognition accuracy of systems that use only single visible images for gender recognition is limited. By using the combination of full body visible and thermal images, we proved that the recognition accuracy is enhanced compared to systems that use only visible or thermal images. In addition, we compared the recognition accuracies when using different feature extraction methods, HoG and MLBP. The experimental results showed that the combination of visible and thermal images produced better recognition accuracy regarding feature extraction methods.

In future work, we plan to enhance the recognition accuracy of this method by counteracting the negative effects such as image’s quality and the effects of background, shadow *etc.* In addition, we plan to collect a larger database for experiments and evaluate the recognition performance using different recognition methods.
